# Expression of p53, Ki-67, sox-2, oct-4, and NANOG in the tongue and trachea of Swiss mice exposed to narghile smoke

**DOI:** 10.1590/1678-7765-2025-0839

**Published:** 2026-05-25

**Authors:** Sarah Freygang Mendes Pilati, Filipe Ivan Daniel, Paulo Vinicius Fontanella Pilati, Mariana Hornung Marins, Aline Cristina Batista Rodrigues, Filipe Modolo

**Affiliations:** 1 Universidade Federal de Santa Catarina Florianópolis SC Brasil Universidade Federal de Santa Catarina, Florianópolis, SC, Brasil.; 2 Pontifícia Universidade Católica do Paraná Escola de Medicina e Ciências da Vida Curitiba PR Brasil Pontifícia Universidade Católica do Paraná, Escola de Medicina e Ciências da Vida, Programa de Pós-Graduação em Odontologia, Curitiba, PR, Brasil.

**Keywords:** Narghile, Carcinogenesis, Tongue, Trachea

## Abstract

**Introduction:**

Narghile (waterpipe) tobacco use has increased worldwide, partly due to limited awareness of its harmful effects. Despite the widespread misconception that the water filters toxic substances, the chemical composition of narghile smoke is comparable to that of cigarette smoke. However, its effects on airway tissues remain poorly understood.

**Objective:**

This study aimed to evaluate the histological and immunohistochemical profiles of the tongue and trachea in Swiss mice exposed to narghile smoke.

**Methodology:**

Sixty animals were divided into six groups: a control group (0 days) and experimental groups exposed daily to narghile smoke for seven, 15, 30, 60, and 90 days. After euthanasia, tongue and tracheal tissues were collected for histological (hematoxylin and eosin staining) and immunohistochemical analyses of NANOG, OCT-4, SOX-2 (neoplastic stem cell markers), Ki-67, and p53 in epithelial cells.

**Results:**

Histological evaluation revealed cytological and architectural alterations in the tongue, suggestive of early dysplastic changes. In the trachea, early metaplastic modifications were observed, including cilia loss, epithelial hyperplasia, and mild keratinization. These changes increased with longer exposure duration. Immunohistochemical analysis showed greater expression of NANOG, OCT-4, SOX-2, and p53, peaking at 30 days, whereas Ki-67 expression peaked at 90 days. Elevated p53 levels suggest alterations in cell cycle regulation and apoptosis.

**Conclusion:**

Narghile smoke induced histological and molecular changes in the tongue and tracheal epithelium of mice, indicating a potential carcinogenic effect. Further *in vitro*, *in vivo*, and clinical investigations are warranted to validate these biomarkers and better elucidate the biological effects of narghile smoke exposure.

## Introduction

With the spread of Arab culture to Western countries, certain cultural practices have also disseminated, including tobacco use through the narghile, also known as waterpipe smoking. Narghile consumption has increased among young individuals, partly due to the misconception that the water in the device filters toxic substances from the smoke, making it less harmful to health.^1^ However, the toxic compounds present in narghile smoke are similar to those found in cigarette smoke.^2^

There is limited evidence regarding the effects of exposure to narghile-derived toxins on the airways.^1^ Such toxic compounds are generated during heating and subsequently inhaled, coming into contact with the airway epithelium and potentially inducing pathological changes.^3^ One approach to evaluate the harmful effects of narghile consumption is the histological evaluation of airway tissues after exposure to toxic agents. This can be accomplished by examining tissue morphology in sections stained with hematoxylin and eosin (H&E), as well as by immunohistochemical analysis of proteins associated with carcinogenesis induced by cigarette smoke, including NANOG, OCT-4, SOX-2, Ki-67, and p53.^4^

Several proteins have been extensively studied in association with malignant neoplasms, among which Ki-67 and p53 are the most well established. Ki-67 is a complex nuclear protein associated with cell proliferation and is widely used as a prognostic marker.^5^ Alterations in Ki-67 and p53 expressions may indicate an increased risk of carcinogenesis.^4^ Previous studies have shown that the severity of epithelial dysplasia correlates with the number of epithelial layers expressing p53 and staining intensity. Notably, mutations in the TP53 gene and loss of its function are associated with the development of squamous cell carcinoma.^6^

The development and progression of several neoplasms, including oral squamous cell carcinoma (OSCC) and lung squamous cell carcinoma (LSCC), have been linked to a subpopulation of tumor cells known as neoplastic stem cells (NSCs).^7^ NSC markers have been associated with the initiation of carcinogenesis and resistance to treatment.^7^ These cells are more prevalent at the invasive tumor front, which may explain their association with more aggressive behavior and worse prognosis.^8^ Several studies have identified NSCs based on the expression of embryonic stem cell markers, such as SOX-2, NANOG, and OCT-4, using techniques including mRNA detection in viable cells. Expression of these markers has been associated with worse prognosis and increased tumor growth potential in breast, bladder, lung, and head and neck neoplasms.^7,8^ SOX-2, OCT-4, and NANOG are central in tumor development and regulate their own transcription via positive and negative feedback loops. OCT-4 acts synergistically with SOX-2 to regulate the transcription of genes involved in stem cell self-renewal and pluripotency.^9^ These markers are particularly relevant in the context of narghile exposure, as narghile smoke contains chemicals known to induce chronic inflammation, oxidative stress, and epithelial remodeling—conditions that have been associated with activation of stemness-related pathways, including SOX2, OCT4, and NANOG, in other tobacco-related settings. Although these markers have been extensively studied in carcinogenesis, their expression in tissues exposed exclusively to narghile smoke remains underexplored.^10,11^ Therefore, investigating their modulation in this context may help clarify whether narghile exposure induces early stemness-associated changes.

## Methodology

This descriptive experimental investigation employed a full-body exposure system.^12,13^ The study was approved by the Ethics Committee of the University of Vale do Itajaí (Univali CEUA; Approval No. 063/17). All animal experiments were conducted in accordance with the Animals in Research: Reporting In Vivo Experiments (ARRIVE) guidelines and applicable national legislation governing the care and use of laboratory animals, consistent with the principles of the U.K. Animals (Scientific Procedures) Act 1986, EU Directive 2010/63/EU, and the National Institute of Health Guide for the Care and Use of Laboratory Animals.

Two-month-old female Swiss mice (n = 60), weighing approximately 25 g each, were housed in conventional cages with ad libitum access to food and water and maintained under a 12-hour light/dark cycle with controlled humidity and temperature. The animals were randomly allocated into six groups (n = 10 per group): one control group and five experimental groups exposed to narghile smoke for seven, 15, 30, 60, and 90 days. Each exposure session lasted 30 min.^14^ Sample size was calculated based on an effect size of 0.4, resulting in a total of 60 animals, which were randomly assigned to six groups (n = 10 per group). All mice were matched for species, sex, age, and weight to ensure homogeneity across groups. For narghile exposure, animals in the experimental groups were placed in a sealed translucent glass chamber (175 × 170 × 270 mm) connected to a standard narghile device via a smoke-delivery system that circulated smoke into the chamber.^15^ Conventional narghile tobacco (Mizo, Al Nakhla Tobacco Company – Free Zone SAE, Shibin El Kom, Egypt), containing 0.5% unwashed tobacco, and Bamboo Brasil charcoal (Egitape Import and Export LTDA, São José, Santa Catarina), measuring 2 cm × 2 cm, were used to generate smoke. The smoke dose delivered to the exposed groups was 35 mL every two seconds, followed by 58 seconds of room air,^16^ totaling 210 mL for each mouse. The pump flow rate was manually adjusted to maintain a volume of 530 mL, as specified by the Beirut method. These parameters were chosen because they approximate the average human breathing pattern during narghile use.^12,14^

The duration of smoke exposure was determined based on previous studies evaluating the cardiorespiratory effects of narghile smoke in humans.^17^ Tongue exposure occurred via deposition of smoke components on the animal's fur, followed by ingestion during self-grooming. Animals in the control group were exposed only to room air under the same environmental conditions as the experimental groups and were euthanized at the corresponding time points.

Euthanasia was performed by anesthetic overdose using 50 μL of xylazine (0.23 g/mL) and 210 μL of ketamine (0.1 g/mL) per 10 g of body weight. The tongue and trachea were harvested, fixed in 10% formalin, embedded in paraffin, and sectioned at 3 μm for H&E staining and immunohistochemistry.

Immunohistochemical analysis was performed using a horseradish peroxidase-labeled polymer method. Sections were dewaxed, rehydrated, and treated in 6% H2O2/methanol solution (1:1) for 30 min to block endogenous peroxidase activity. Antigen retrieval was conducted with citrate buffer (pH 6.0) for 30 min in a microwave oven at 1050 W. Nonspecific binding sites were blocked with 5% skim milk in phosphate-buffered saline for 40 min. Primary antibodies were incubated overnight at 4 °C (sources, dilutions, and positive controls for each antibody are shown in Table 1), followed by incubation with the EnVision+ System (Dako Corporation, Carpinteria, CA, USA) and two min of incubation with diaminobenzidine (DAKO Liquid DAB plus, Dako Corporation, Carpinteria). Sections were then counterstained with Harris hematoxylin. After dehydration and clearing, slides were mounted using Entellan (Merck, Darmstadt, Hessen, Germany). Positive controls were included for all antibodies (Table 1), and negative controls were obtained by omitting the primary antibody. For each sample, field locations were standardized to ensure uniform spatial distribution. In the tongue, six equidistant areas were evaluated (two on the dorsum, two on the ventral surface, and one on each lateral border). In the trachea, four equidistant areas around the lumen were analyzed at 200 × magnification using a light microscope equipped with a camera (Canon A620, San Jose, CA, USA). Histopathological evaluation was performed according to the World Health Organization classification of dysplasia. Immunoexpression was determined by the presence of brown staining in epithelial cells.

**Table 1:** Antibodies, clones, dilution, and positive controls used

Antibody	Clone	Dilution	Positive control
**Nanog**	TA306915	1:100	Spleen
**OCT-4**	TA354593	1:100	Oral squamous cell carcinoma
**SOX-2**	TA321560	1:100	Thyroid
**Ki-67**	SP6	1:300	Oral squamous cell carcinoma
**p53**	SAB4504499	1:300	Oral squamous cell carcinoma

Nuclear chromogen presence was used to determine antibody immunopositivity in each sample, and only the percentage of positive nuclear cells among all counted cells was analyzed.^18,19^ Image acquisition and immunoreactivity analysis were performed by an examiner blinded to group allocation. The examiner underwent calibration, and intra-observer reliability was confirmed with an intraclass correlation coefficient greater than 90%. Reactions were evaluated by a single blinded and calibrated investigator (kappa > 0.8) using the ImageJ 1.410 software (National Institute of Health, Bethesda, Maryland, USA).

Statistical analyses were performed using the SPSS software (version 11; SPSS Inc., USA). Nonparametric statistical tests were applied, since the data showed a non-Gaussian distribution. Nuclear immunopositivity for each protein was compared among groups and according to histopathological classification using the Kruskal–Wallis test. Correlations between protein expression levels were assessed using Spearman's correlation coefficient. Statistical significance was set at *p* < 0.05.

## Results

### Tongue

In the H&E-stained slides, the control, seven-day, and 15-day exposure groups exhibited normal tongue epithelium. At 30 days of exposure, slight epithelial alterations were observed, which became more pronounced at 60 and 90 days. Cytological and architectural alterations included epithelial acanthosis, focal hyperchromasia, mild keratinization, drop-shaped rete ridges, loss of basal cell polarity, an increased number of mitotic figures, and chronic inflammation in the lamina propria. Accordingly, the 30-, 60-, and 90-day groups exhibited focal alterations suggestive of epithelial dysplasia.

Exposure to narghile smoke resulted in reduced expression of NSC markers in the seven-day group, with a gradual increase from 15 to 30 days (peak), a subsequent decrease at 60 days, and a new increase at 90 days (Table 2).

**Table 2:** Median positive cell count per field and interquartil range of Nanog, OCT-4, SOX-2, Ki-67 and p53 in tongue epithelium across different durations of narghile smoke exposure

		Control	07 days	15 days	30 days	60 days	90 days
		n (10)	n (10)	n (10)	n (10)	n (10)	n (10)
**Tongue**	Median number of NANOG + cells per field	7.23^ac^	0.78^c^	31.87^ab^	61.67^b^	22.04^abc^	41.39^ab^
	Interquartil range	24.42	2.38	44.11	29.27	17.92	24.86
	Median number of OCT-4 + cells per field	13.29^ab^	2.43^a^	18.59^ab^	42.19^b^	7.14^a^	40.09^b^
	Interquartil range	20.17	7.71	18.5	51.08	10.22	22.71
	Median number of SOX-2 + cells per field	1.41^a^	0.61^a^	3.66^ab^	27.03^b^	1.79^ab^	6.19^ab^
	Interquartil range	2.82	2.59	23.82	26.28	6.31	7.95
	Median number of Ki67 + cells per field	0.15^ab^	0.10^a^	0.58^abc^	0.40^abc^	0.75^bc^	1.40^c^
	Interquartil range	0.35	0.39	1.28	0.41	0.87	2.51
	Median number of p53 + cells per field	0.26^ac^	0.10^a^	2.14^bc^	3.12^b^	2.84^bc^	3.10^b^
	Interquartil range	0.47	0.38	2.46	2.1	3.08	2.32

Note: Data are expressed as Median and Interquartile. Statistical analysis was performed using the Kruskal-Wallis test followed by the Bonferroni *post-hoc*. Different letters indicate statistically significant differences (*p* < 0.05).

NANOG expression decreased from the control to day seven (0.78%). The seven-day group differed significantly from the 15-, 30-, and 90-day groups (*p* = 0.003, *p* < 0.001, *p* < 0.001, respectively), with expression increasing progressively from day 15 and peaking at day 30 (61.67%). OCT-4 expression also decreased in the seven-day group (2.43%) and increased progressively in the 15- and 30-day groups (18.59% and 42.19%, respectively). Statistically significant differences were observed between the seven- and 30-day groups (*p* = 0.00) and between the seven- and 90-day groups (*p* = 0.002).

**Figure 1:**
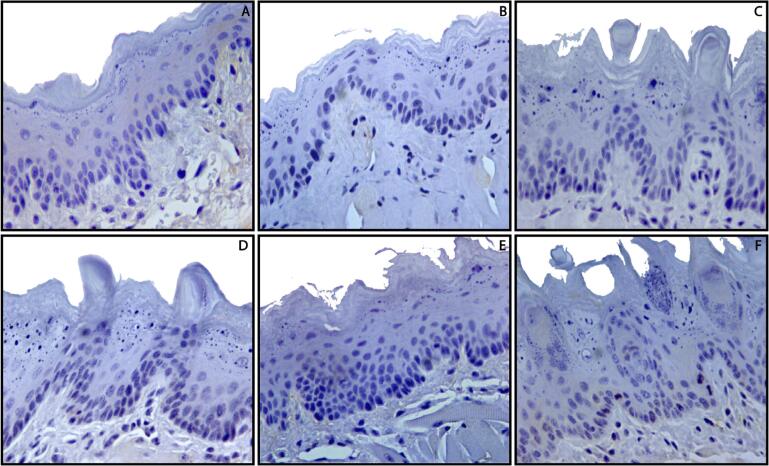
Ki-67 immunoexpression in the tongue epithelium of Swiss mice exposed to narghile smoke for different durations

SOX-2 expression was similar in the control (2.82%) and the seven-day groups (2.59%), increased at 15 days (23.82%), and peaked at 30 days (26.28%). A significant difference was observed between the 30-day group and both the control and seven-day groups (*p* = 0.016 and *p* = 0.002, respectively). Ki-67 expression increased from 15 days (0.58%) to 60 days (0.75%) and 90 days (1.40%). A statistically significant difference was found only between the control and 90-day groups (*p* = 0.004) (Figure 1).

**Figure 2:**
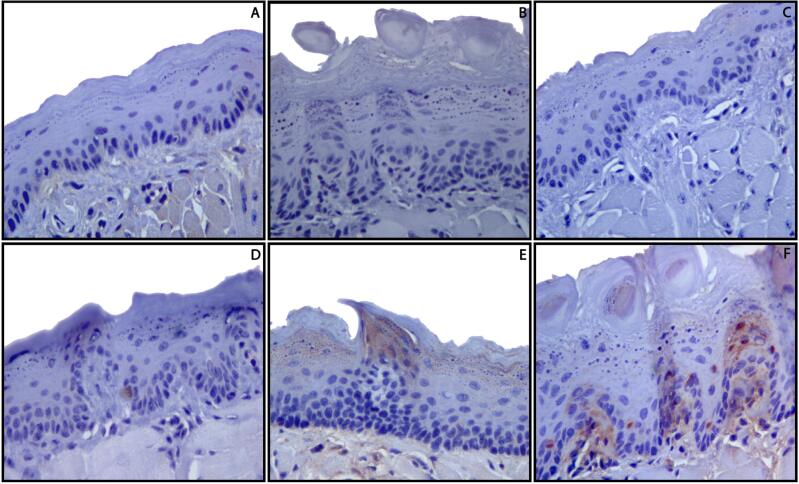
p53 expression in the tongue epithelium of Swiss mice exposed to narghile smoke for different durations

p53 expression was comparable between the control (0.26%) and the seven-day group (0.10%). A slight increase was observed at 15 days (2.14%), with higher expression at 30 and 90 days (3.12% and 3.10%, respectively). Statistically significant differences were found between the control group and both the 30-day (*p* = 0.003) and 90-day (*p* = 0.016) groups (Figure 2).

Spearman's correlation test revealed moderate correlations among NANOG, OCT-4, and SOX-2 expression levels. Significant correlations were observed between NANOG and OCT-4 (R = 0.565; *p* < 0.001), NANOG and SOX-2 (R = 0.596; *p* = 0.000), and OCT-4 and SOX-2 (R = 0.512; *p* < 0.001). Additionally, moderate and significant correlations were observed between p53 and the NSC-associated proteins NANOG (R = 0.500; *p* < 0.001) and SOX-2 (R = 0.459; *p* < 0.001). A significant but weak correlation was observed between p53 and OCT-4 (R = 0.373; *p* = 0.003), as well as between p53 and Ki-67 expression in the tongue (R = 0.392; *p* = 0.02).

### Trachea

H&E staining revealed normal histological features in the control, seven-day, and 15-day exposure groups. In the 30-day group, focal areas showed ciliary loss and/or basal cell hyperplasia within the pseudostratified epithelium. In the 60- and 90-day groups, areas of stratified squamous epithelium exhibited extensive ciliary loss and focal mild hyperkeratosis, indicative of early metaplasia. A reduction in connective tissue thickness and the presence of chronic inflammatory infiltrate were also observed (Table 3).

**Table 3:** Median positive cell count per field and interquartile range of NANOG, OCT-4, SOX-2, Ki-67 and p53 in tracheal epithelium across different durations of narghile smoke exposure.

		Control	07 days	15 days	30 days	60 days	90 days
		n (10)	n (10)	n (10)	n (10)	n (10)	n (10)
**Trachea**	Median number of NANOG + cells per field	41.76^a^	4.64^b^	25.40^ab^	37.68^a^	11.16^ab^	42.55^a^
	Interquartil range	23.93	13.83	10.23	37.23	24.25	33.12
	Median number of OCT-4 + cells per field	49.14^a^	6.97^b^	26.68^ab^	26.51^ab^	6.19^b^	30.79^ab^
	Interquartil range	28.31	6.25	21.19	17.59	12.9	19.81
	Median number of SOX-2 + cells per field	2.63^a^	4.98^a^	13.19^a^	2.76^a^	0.30^a^	2.76^a^
	Interquartil range	29.66	7.36	29.49	35.54	7.88	23.95
	Median number of Ki-67 + cells per field	0.00^a^	0.39^a^	2.67^ab^	5.56^b^	1.01^a^	2.46^ab^
	Interquartil range	1.13	0.96	3.38	6.23	2.05	4.77
	Median number of p53 + cells per field	0.78^a^	1.18^ab^	1.67^ab^	2.29^ab^	2.31^b^	1.42^ab^
	Interquartil range	1.31	1.33	3.68	1.68	4.72	3.47

Note: Data are expressed as Median and Interquartile Range. Statistical analysis was performed using the Kruskal-Wallis test followed by the Bonferroni *post-hoc*. Different letters indicate statistically significant differences (p < 0.05).

NANOG expression decreased in the seven-day group (4.64%), increased at 15 and 30 days (25.40% and 37.68%, respectively), decreased at 60 days (11.16%), and increased again at 90 days (42.55%), similar to the expression pattern observed in the tongue. A statistically significant difference was observed between the control and seven-day groups (*p* = 0.005). OCT-4 expression decreased in the seven-day group (6.97%), increased at 15 and 30 days (26.68% and 26.51%, respectively), decreased at 60 days (6.19%), and increased again at 90 days (30.79%). A statistically significant association was found between the control and seven-day groups (*p* < 0.001) and between the control and 60-day groups (*p* < 0.001). SOX-2 expression increased at seven (4.98%) and 15 days (13.19%) and decreased at 30, 60, and 90 days (2.76%, 0.30%, and 2.76%, respectively), with no statistically significant differences. Low Ki-67 expression was observed in the narghile-exposed groups, with a slight increase over time and peaking at 30 days (5.56%). Statistically significant differences were observed between the control and 30-day groups (*p* < 0.001). p53 expression was elevated in the 30-day group, but the increase was statistically significant only between the control and 60-day groups (*p* = 0.029).

Spearman's correlation test showed a moderate correlation between OCT-4 and NANOG (R = 0.513; *p* < 0.001) and a weak correlation between OCT-4 and SOX-2 (R = 0.313; *p* = 0.017). No correlation was observed between SOX-2 and NANOG (R = 0.174; *p* = 0.192). Furthermore, a statistically significant but weak correlation was found between p53 and Ki-67 (R = 0.369; *p* = 0.004).

## Discussion

In this study, cell proliferation and apoptosis were evaluated using Ki-67 and p53 expression, along with NSC markers NANOG, SOX-2, and OCT-4. NSC markers are associated with the presence of pluripotent cells, and aberrant expression of their isoforms and pseudogenes are significant in tumor carcinogenesis and metastasis; however, the underlying mechanisms remain poorly understood.^20^

Our results showed a gradual increase in NSC-associated markers in the lingual epithelium, with peak NANOG, OCT-4, and SOX-2 expression at 30 days of exposure. Early upregulation of these proteins may suggest tissue renewal and protection against cell differentiation, indicating a compensatory response to injury caused by narghile smoke.^20^ Liu, Yu, and Liu^20^ (2013) reported that these markers not only maintain the self-renewal and pluripotency properties of pluripotent cells but also prevent inappropriate cell division.^2^ Thus, their expression in the lingual epithelium may indicate early tissue damage induced by narghile smoke. Moreover, increased OCT-4 and SOX-2 expression has been linked to early stages of malignancy, which is consistent with the progressive increase observed up to 30 days in this study. However, the subsequent decrease at 60 and 90 days may indicate cellular adaptation to chronic chemical exposure.^21^ NSC markers were also detected in the control group, possibly reflecting high tissue renewal rates of lingual dorsum epithelial cells in rodents.^22^

Significant correlations were observed among NSC markers in the tongue, which is particularly relevant to this study, as the co-expression of these three markers has been associated with tumor genesis, progression, and differentiation of neoplastic cells, including OSCC.^7,23^ These findings suggest that narghile smoke may upregulate NSC expression and increase the risk of malignant transformation. The moderate correlations observed indicate that these NSC-associated proteins may act together in response to narghile-induced injury. Increased expression of SOX-2, OCT-4, and NANOG correlated with the presence of NSCs, as well as with a worse prognosis in OSCC. Notably, double-positive OCT-4/SOX-2 cells have been detected in potentially malignant oral mucosa lesions.^8,24^

A significant increase in cell proliferation was observed in the 60-day group, as indicated by Ki-67 expression, which has been associated with dysplastic changes in the oral cavity and with more severe dysplastic classifications. Although epithelial dysplasia was not observed, Ki-67 expression may suggest altered proliferative activity in response to narghile-induced injury.^25^ Notably, p53 expression has been reported in narghile users even in histologically normal tissues,^26^ which was also observed in our study, particularly in the 30-day group. This indicates that components of narghile smoke may modulate p53 expression. Even when evaluated in isolation, altered p53 expression has been associated with carcinogenesis, worse prognosis, and greater recurrence of lesions.^6^ In our study, a weak correlation was observed between p53 and Ki-67 expression in the tongue. Increased expression of both proteins has been associated with higher grades of dysplasia and worse lesion prognosis, supporting their combined expression as a potential marker of carcinogenesis.^4,23^ These findings indicate that narghile exposure may interfere with both apoptotic and cell proliferative pathways, potentially contributing to the development of oral malignancies, including OSCC. Additionally, correlations were observed between NANOG, OCT-4, SOX-2, and p53 expression in the tongue. OCT-4 is known to contribute to NSC survival by inhibiting apoptosis, partly via activation of the OCT-4/TCL1/AkT1 pathway. This pathway interacts with p21, which is associated with p53, thus explaining the correlation observed when p53 is mutated.^18,27,28^ Furthermore, an association was observed between increased NANOG and p53 expression. According to Lee, et al.^29^ (2015), increased NANOG expression and mutant p53 are significantly associated with clinically advanced stage tumors, positive cervical lymph node metastasis, high histological grade tumors, and poor overall survival in OSCC. Thus, the moderate correlation between these markers may reflect more advanced lesions, demonstrating narghile-induced tissue injury.

The use of conventional cigarettes triggers a hyperproliferative response, resulting in progressive and potentially malignant epithelial alterations ranging from squamous metaplasia to dysplasia.^30^ Dysplastic changes were not observed in this study. However, early metaplastic changes were identified, including loss of cilia, epithelial hyperplasia, and focal hyperkeratosis, indicating that narghile use may induce alterations similar to those caused by cigarettes, as also observed by Van den Berg, et al.^31^ (2007) and Shraideh and Najjar^13^ (2011). It should be noted that dysplastic changes are uncommon in conventional smokers who progress to SCCP, with metaplasia being an important feature for early diagnosis.^32^ These initial epithelial alterations may be closely associated with inflammatory and oxidative stress pathways involved in tobacco-related tissue damage. Although such molecular pathways were not investigated in this study, future research evaluating inflammatory mediators and related biomarkers may help further elucidate the biological mechanisms underlying narghile-induced epithelial injury.

In the trachea, NANOG and OCT-4 expression peaked in the 90-day group. SOX-2 expression was the only marker that did not show a statistically significant difference and remained lower over time. Its low expression in the trachea is consistent with the findings of Kim, et al.^30^ (2016), who reported a possible decrease in SOX-2 promoter activity during the progression of tracheal epithelial alterations.^30^ Another study demonstrated that increased SOX-2 expression in the trachea and lungs may occur later, taking up to 34 weeks in mice.^32^

The expression of NSC-associated proteins in the trachea of the control group was expected, as previous studies have shown that murine airways endogenously express these markers.^18,33^

In the trachea, OCT-4 expression correlated with both NANOG and SOX-2 expression, which may indicate early epithelial alterations, as the expression of these NSC markers regulates epithelial–mesenchymal transition, controls tumor initiation capacity, and is associated with worse prognosis.^34^

The expression of p53 in the trachea gradually increased and reached statistical significance in the 60-day group, consistent with previous reports indicating that histological changes in the respiratory epithelium associated with p53 may be an early marker of squamous cell carcinoma, especially in high-risk populations.^35,36^ Increased p53 expression indicates alterations in cell cycle regulation and apoptosis in tissues exposed to narghile smoke, with levels increasing over time and peaking in the 60-day group, indicating worsening of tissue injury. Furthermore, previous studies have shown that airway epithelial hyperplasia and early neoplastic stages are associated with alterations in p53 expression.^37^ Interestingly, in this study, narghile-induced changes appeared earlier than those reported for cigarette exposure. Magnani, et al.^38^ (2015), who exposed guinea pigs to conventional cigarettes for 260 days, did not report p53 expression in the tracheal epithelium, indicating that narghile smoke may induce more pronounced alterations than conventional cigarettes.

In the trachea, Ki-67 expression was detected with greater intensity than in the tongue. This may be explained by the fact that the tracheal epithelium is nonkeratinized and pseudostratified and, therefore, more susceptible to external environmental aggressions compared with the tongue epithelium.^35^ Ki-67 expression increased from the 30-day group onward, indicating increased cell proliferation. This proliferative response has been associated with epithelial hyperplasia and may represent a protective mechanism against tissue injury.^39^ Increased Ki-67 expression has been linked to the development of airway carcinomas and the acquisition of an invasive phenotype.^40,41^ The results of this study are consistent with previous reports in conventional cigarette smokers, in whom increased Ki-67 expression has been detected in the airways.^39^ Moreover, the correlation between both markers, as observed in our study, has been associated with worse prognosis and more severely classified airway lesions.^42^

The effect of female sex hormones, especially estrogens, on the development of neoplasms in rodent models has been widely reported in the scientific literature. This suggests that ovarian hormones may modulate both cell proliferation and cancer-related inflammatory pathways.^43^ Female rodents are often excluded from studies on OSCC due to hormonal variability, which may introduce bias by either amplifying or reducing the biological response in these models. Nevertheless, the inclusion of female animals is crucial to improve the understanding of disease mechanisms in this understudied portion of the population.

## Conclusion

This study identified histological and immunohistochemical alterations in the tongue and tracheal tissues of mice exposed to narghile smoke. In the tongue, only mild cytological and architectural changes suggestive of early dysplasia were observed, whereas in the trachea, ciliary loss, epithelial hyperplasia, and discreet hyperkeratinization indicated initial metaplastic responses. These alterations intensified gradually with prolonged exposure.

Immunohistochemical findings revealed time-dependent variations in NSC markers, p53, and Ki-67 expression. In the tongue, NSC markers and p53 peaked at day 30, whereas Ki-67 reached its highest expression at day 90. In the trachea, NANOG expression was greatest at day 90, SOX-2 showed variation over time, Ki-67 peaked at day 30, and p53 at day 60. These patterns suggest modulation of pathways involved in cell-cycle regulation and proliferation.

Moderate correlations between p53 and Ki-67, as well as between NSC-related proteins and p53, indicate a potential interplay between proliferative activity and stem cell-associated pathways. However, the observed changes appear to represent adaptive or early preneoplastic responses. Therefore, these findings should be interpreted with caution, as they do not provide definitive evidence of carcinogenic potential.

## Data Availability

The data supporting the findings of this study are available in the SciELO Data repository - doi: 10.48331/SCIELODATA.36CVQQ.
